# Olaparib-induced pseudoporphyria in a patient with ovarian cancer

**DOI:** 10.1016/j.jdcr.2023.07.011

**Published:** 2023-07-21

**Authors:** Abigale Clark, Amanda S. Weissman, Arthur Neil Crowson, Jason Hirshburg

**Affiliations:** a Department of Dermatology, University of Arkansas for Medical Sciences, Little Rock, Arkansas; b Department of Dermatology, University of Oklahoma Health Science Center, Oklahoma City, Oklahoma; c Department of Dermatology and Pathology Laboratories Inc, University of Oklahoma, Tulsa, Oklahoma

**Keywords:** bullous, Olaparib, phototoxicity, photodermatoses, Pseudoporphyria

## Introduction

Pseudoporphyria is a rare and poorly understood bullous photodermatosis associated with certain phototoxic medications, UV radiation, hemodialysis, and indoor tanning bed use. Although pseudoporphyria closely resembles porphyria cutanea tarda (PCT) from a clinical and histologic standpoint, there are characteristically no laboratory abnormalities.^1^ Patients with pseudoporphyria typically present with skin fragility, vesicles, bullae, milia, and/or scarring in photoexposed areas. Although involvement of the dorsal aspect of the hands is most common, the characteristic lesions of pseudoporphyria can also be found on the extensor legs, fingers, upper portion of the chest, and face.^1^^,^^2^

Clinical history-taking is essential because patients with pseudoporphyria will often have a history of chronic renal disease, indoor tanning bed use, or recent exposure to phototoxic medications.^1^ Although many medications have been reported to cause pseudoporphyria, propionic acid derivative nonsteroidal anti-inflammatory drugs, such as naproxen, are the most reported culprit.^3^ Poly-ADP ribose polymerase (PARP) inhibitors, such as olaparib, are not a commonly known cause of pseudoporphyria.

## Case report

A 71-year-old Filipino woman with ovarian cancer and a long-standing history of psoriasis treated with topical medications presented with a 1-year history of a pruritic, blistering rash that began shortly after beginning olaparib for the treatment of her ovarian cancer. The rash occurred intermittently on her face, chest, upper extremities, and lower extremities, with lesions beginning as erythematous macules that transformed into vesicles and eventually became firm papules. Each lesion cycle typically lasted one week before resolving. She reported minor itch relief with application of her psoriasis medications—clobetasol ointment 0.05% and tacrolimus ointment 0.1%, applied twice daily as needed. She denied any inciting factors or triggers with exception of starting olaparib therapy several weeks prior to the rash developing.

**Fig 1:**
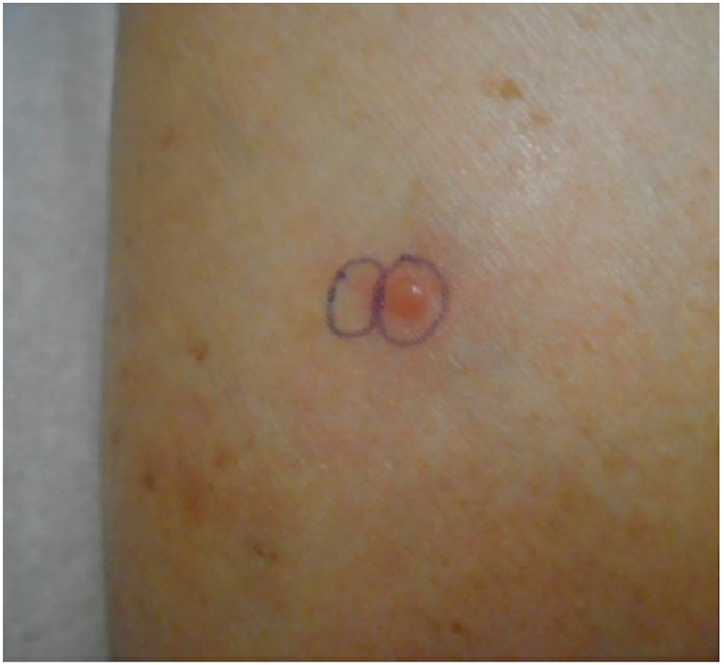
An erythematous vesicle on the left medial shin.

Physical examination revealed scattered erythematous macules and firm papules present on the nose, right ankle, right dorsal aspect of the foot, and bilateral lower extremities. One erythematous vesicle was present on the left medial shin (Fig 1). Psoriatic plaques, consistent with her preexisting diagnosis of psoriasis, were also present on the bilateral lower extremities. Two 4-mm punch biopsies were taken from the medial left shin, including a perilesional punch biopsy that was sent for direct immunofluorescence (DIF) testing. The patient was prescribed hydroxyzine 25 to 50 mg nightly as needed for itch and sleep.

**Fig 2:**
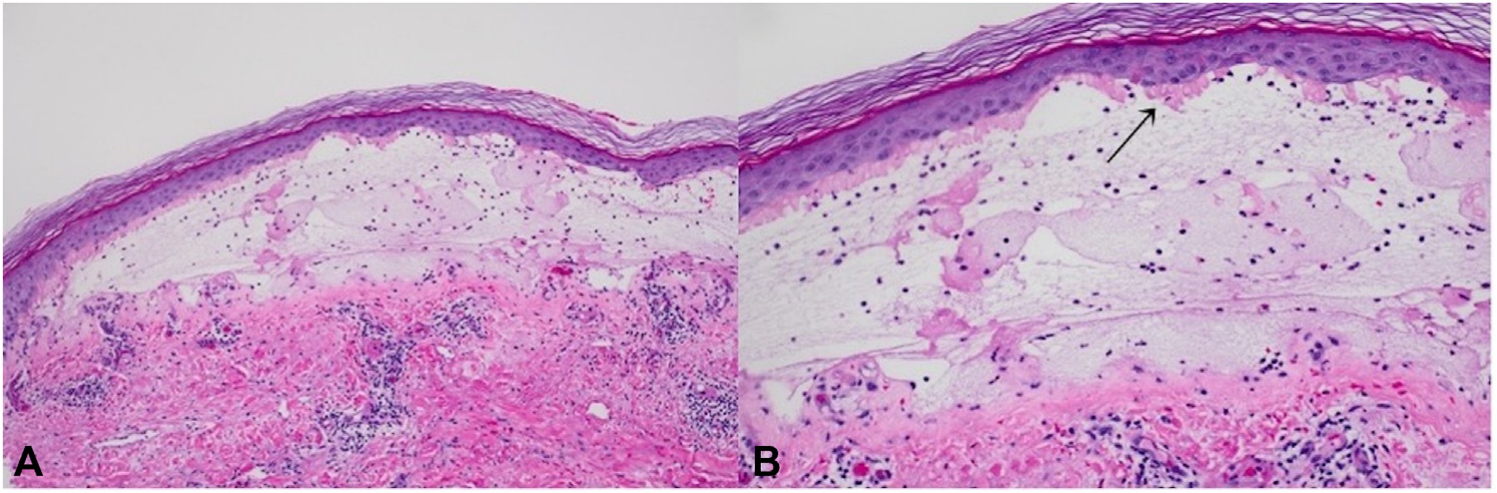
Hematoxylin-eosin staining of punch biopsy taken from left medial shin (10× magnification) showing a subepidermal blister. **A,** The cavity contains mononuclear cells and granulocytes—mainly neutrophils but also eosinophils. **B,** The blister roof comprises epidermis showing degenerative features and there are “caterpillar bodies” adherent to the undersurface of the epidermis (*arrow*). (**A** and **B**, Hematoxylin-eosin stain; original magnification: 10×.)

**Fig 3:**
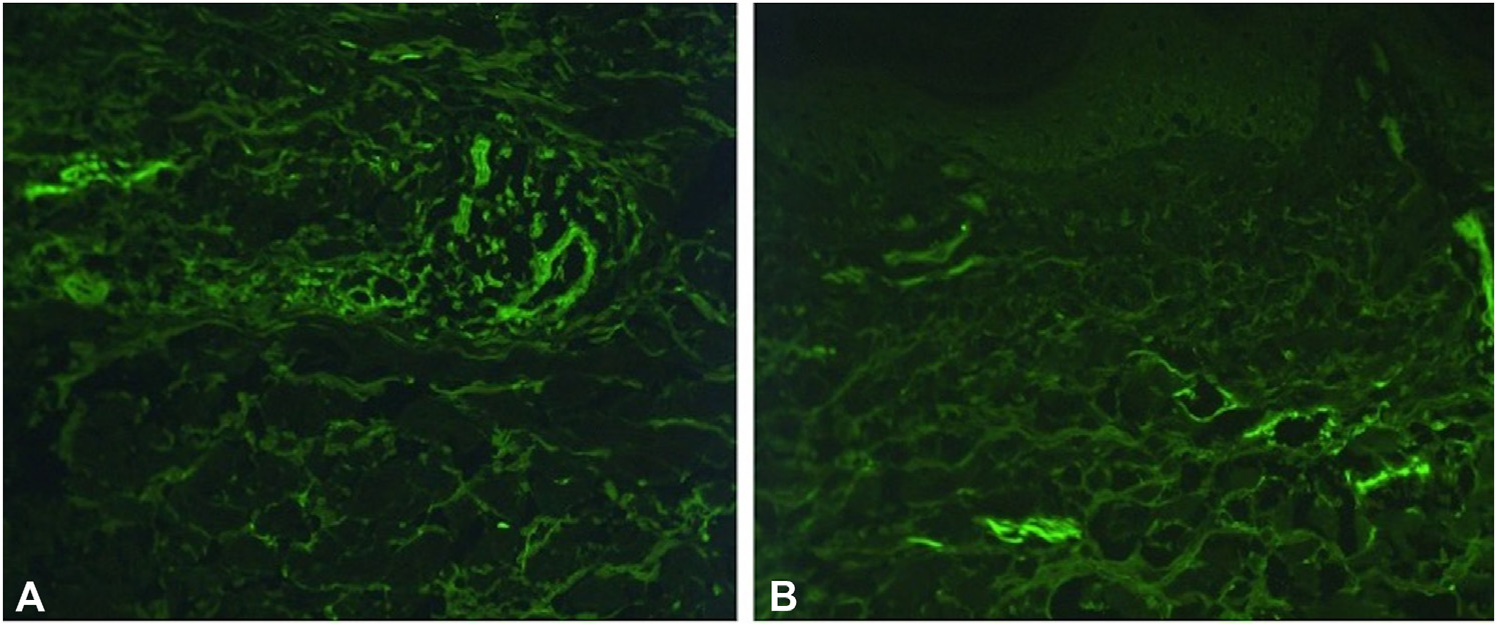
Positive direct immunofluorescence of biopsy taken from left medial shin. Homogenous deposition of IgG (**A**) and IgA (**B**) in thick-walled blood vessels. Tissue was negative for immunoglobulin M, C1q, C3, and fibrin.

Results of the punch biopsy (Fig 2*A*, *B*) were consistent with a subepidermal bullous disorder, showing a subepidermal blister with sparse mononuclear cells and granulocytes, in addition to “caterpillar bodies” adherent to the undersurface of the epidermis. DIF test findings were positive for homogenous deposition of IgG and IgA in thick-walled blood vessels (Fig 3*A*, *B*), all of which suggested a diagnosis of PCT vs pseudoporphyria.

Further workup to determine the diagnosis revealed total plasma porphyrin level within normal limits. Given the patient’s clinical presentation, histologic findings, and normal laboratory results, a diagnosis of pseudoporphyria was made. The temporal association of beginning olaparib therapy and development of the rash suggests drug-induced pseudoporphyria as the most likely explanation for this patient’s presentation. To further support this conclusion, the patient’s rash persisted for the duration of therapy with olaparib and resolved shortly after discontinuing the medication.

## Discussion

Although considered rare in the United States, porphyria constitutes a group of 9 disorders characterized by abnormal functioning of the heme biosynthesis pathway. Each of these diseases involves a unique enzymatic abnormality that ultimately leads to the accumulation of porphyrins and other precursors of heme in the bloodstream. The accumulation of these chemicals leads to acute neurologic manifestations as seen in acute intermittent porphyria or phototoxicity as seen in PCT. In PCT, phototoxicity occurs due to the accumulation of porphyrin in the skin or dermal blood vessels and is characterized by the formation of vesicles or bullae on photoexposed skin.^4^ In addition, patients with PCT classically have elevated total plasma porphyrin levels.^5^

When a patient presents with clinical and histologic features consistent with PCT but without accompanying laboratory abnormalities, a diagnosis of pseudoporphyria is made. Histologically, the findings in pseudoporphyria are very similar to PCT and show a subepidermal blister with sparse inflammatory infiltrate. “Caterpillar bodies,” seen microscopically as linear eosinophilic globules comprising necrotic keratinocytes embedded in a thickened basement membrane zone adherent to the undersurface of the detached epidermis, are a characteristic finding in PCT. Results of DIF testing are similar in both conditions, with deposition of complement and immunoglobulins, particularly IgG, surrounding blood vessels.^6^

Because of the major overlap in presentation, differentiating between PCT and pseudoporphyria relies largely on laboratory testing and clinical history. Total plasma porphyrin levels will classically be elevated in patients with PCT, whereas plasma porphyrin abnormalities will be absent in pseudoporphyria. Although previous literature has documented pseudoporphyria in association with nonsteroidal anti-inflammatory drugs, antibiotics, diuretics, retinoids, and various systemic medications, a case of olaparib-associated pseudoporphyria is exceedingly rare.

Olaparib is an oral PARP enzyme inhibitor that induces cell death in breast cancer gene (BRCA1/2) deficient tumor cells through the formation of double-stranded DNA breaks. This drug is indicated for use in patients with advanced ovarian cancer and certain BRCA-mutated ovarian cancers. Common adverse reactions of olaparib and other PARP inhibitors include hematologic toxicities, gastrointestinal adverse events, fatigue, and renal toxicity.^7^ Although dermatologic adverse reactions have been reported in association with each of the approved PARP inhibitors, a mild rash is most common.^8^ In one study, photosensitivity reactions were documented in 17% of patients taking rucaparib, a PARP inhibitor that is closely related to olaparib. Importantly, photosensitivity reactions were the most common cutaneous reactions observed in this study.^9^ It is believed that the phototoxicity induced by rucaparib can be attributed to its ability to induce photosensitized damage to lipids, proteins, and DNA.^10^ Although rucaparib is currently the only PARP inhibitor shown to induce photosensitized damage to biomolecules in vitro, it is reasonable to postulate that olaparib could have similar effects.

This case shows the potential for phototoxic effects when taking olaparib and highlights the importance of discussing this potential adverse event with patients before initiating therapy. In addition, it is imperative for clinicians to counsel patients on the importance of sun protection with sunscreen, hats, and sun-protective clothing when taking these drugs.

## Conflicts of interest

None.
